# Comparison of a low carbohydrate intake and standard carbohydrate intake on refeeding hypophosphatemia in children and adolescents with anorexia nervosa: a pilot randomised controlled trial

**DOI:** 10.1186/s40337-021-00519-0

**Published:** 2022-04-12

**Authors:** Kellie Draffin, Jessica Hamilton, Shea Godsil, Suba Rudolph, Tim Crowe, Richard Newton

**Affiliations:** 1 Austin Health, Melbourne, Australia; 2 Peninsula Health, Melbourne, Australia

**Keywords:** Refeeding syndrome, Anorexia nervosa, Hypophosphatemia, Refeeding, Aggressive feeding, Adolescent, Nutrition, Carbohydrate

## Abstract

**Background:**

Nutritional rehabilitation for patients with anorexia nervosa involves balancing the need for weight gain whilst mitigating the risk of refeeding syndrome. Graded caloric increases and restriction of calories from carbohydrate have been used to minimise the risk of developing refeeding hypophosphatemia. There is little evidence to support the recommended nutrient composition, specifically the recommended carbohydrate intake that is safe in this population. The aim of this pilot study was to compare the effect of a low and a standard carbohydrate feeding protocol on serum phosphate levels in children and adolescents with anorexia nervosa.

**Methods:**

A pilot study of 23 children and adolescents with anorexia nervosa admitted for medical stabilisation to the adolescent ward of a tertiary hospital was undertaken. Participants were commenced on an oral feeding protocol and were randomly allocated to isocaloric meal plans that were either low carbohydrate (< 40% total energy from carbohydrate) or standard carbohydrate (50–60% total energy from carbohydrate). Serum phosphate levels were monitored daily across the first week and twice weekly thereafter. Clinical status, including weight gain, was monitored throughout admission.

**Results:**

52% (n = 12) of participants were allocated to the low carbohydrate group and 48% (n = 11) were allocated to the standard carbohydrate group. No patients in either of the diet groups developed refeeding hypophosphatemia in the first seven days of admission. Weight gain during the first week was significantly higher in the standard carbohydrate diet (1.4 kg/wk ± 0.5) compared to the low carbohydrate diet (0.6 kg/wk ± 0.9), *p* value 0.03. Participants from both diet groups were largely orally fed with less than 10% of the total number of meals and/or snacks across both groups provided as nutrition supplement drinks, either orally or enterally.

**Conclusion:**

This pilot study supports that a standard carbohydrate intake (providing 50–60% of total energy from carbohydrate) optimises nutritional rehabilitation without increasing the risk of refeeding hypophosphatemia in adolescent inpatients with anorexia nervosa.

CTN: ACTRN12621000300875.

**Plain English Summary:** People with eating disorders who are underweight or malnourished, such as patients with anorexia nervosa, are at risk of refeeding syndrome when they receive treatment and return to regular eating. Refeeding syndrome may cause fluid and electrolyte shifts. This can occur as a result of the reintroduction of carbohydrates, and can have potentially life-threatening consequences if not managed appropriately. Refeeding hypophosphatemia is one of the early markers of refeeding syndrome. This study compared patients who were provided a low carbohydrate diet (40% total energy from carbohydrate) to those who were provided a standard carbohydrate diet (50–60% total energy from carbohydrate) to see if patients from either group were more at risk of developing refeeding syndrome. No patients in either of the diet groups developed refeeding hypophosphatemia. This pilot study may help to ensure that when patients get treated for their eating disorder in hospital, they can return to a normal diet as soon as possible with close medical monitoring.

## Introduction

Nutritional rehabilitation is integral in the treatment of anorexia nervosa (AN) to medically stabilise the patient and reverse the effects of malnutrition [1]. The provision of adequate nutrition to achieve weight restoration and medical stability must be balanced against managing the risks of refeeding syndrome (RFS). RFS can result in serum electrolyte shifts and fluid imbalance due to metabolic changes following the reintroduction of nutrition in malnourished patients [2]. This can result in potentially life-threatening consequences for the patient across a range of organ systems including the neurological, cardiovascular, respiratory, gastrointestinal, haematological and musculoskeletal systems [2, 3]. Of particular concern is the risk of cardiac arrhythmias resulting in sudden death [3]. Refeeding hypophosphatemia (RH) is an abnormally low phosphate level in the blood that may result following reintroduction of nutrition [4]. RH is defined as < 0.95 mmol/L (< 2.94 mg/dL) in patients under 16 years and < 0.87 mmol/L (< 2.69 mg/dL) in patients > 16 years as per local hospital guideline. RH occurs early in RFS and is commonly used as an indicator for development of RFS [2]. The risk of hypophosphatemia during nutritional rehabilitation in adolescent inpatients with AN has been estimated to be around 14% [4]. The potential for developing RFS is the greatest during the initial 72 h following commencement of nutritional rehabilitation [5]. Patients with AN are vulnerable to the development of RFS due to their low body stores of electrolytes from chronic starvation and the degree of malnutrition [6, 7]. Patients who are less than 70% of their expected body weight, have had prolonged inadequate intake or have hypophosphatemia on commencing nutritional rehabilitation are considered to be at high risk [2, 7, 8].

Guidelines for the prevention and management of RFS in hospitalised patients recommend providing between 40 and 60% of total energy from carbohydrate sources, alongside graded increases in caloric intake from 5 to 25 kcal/kg/day depending on level of RFS risk [2]. These guidelines however exclude patients with AN as they are believed to be different from a pathophysiological perspective [1, 2].

In patients hospitalised with AN, a graded approach, commencing around 1250 kcal/day with slow advancement in calories, has historically been used to mitigate the risk of developing RFS [8]. However, underfeeding and overly cautious approaches to nutritional rehabilitation in this patient group may delay resolution of serious medical complications and lead to poor weight gain or even weight loss due to the hyper-metabolic state. This has been identified as a contributor to prolonged length of stay and potentially fatal outcomes [7]. In the longer term, weight restoration is critical in overcoming the physical complications caused by AN, including amenorrhoea, pubertal delay, reduced bone density, and impaired cognition [1, 6, 8, 9]. For hospitalised patients with AN, the rate and amount of weight gain has been shown to predict positive outcomes at 1 year follow-up [10]. Following the systematic review by Garber et al. which evaluated approaches to refeeding patients with AN across 27 studies, the safety and benefit of commencing at a higher caloric intake with more rapid increases for hospitalised patients with AN has allowed for more aggressive feeding protocols to be implemented [1, 11, 12].

Current guidelines advise restricting calories from carbohydrates and including foods rich in phosphate during nutritional rehabilitation in order to avoid RFS in patients with AN [7]. However, there have been few studies that have investigated the effect of nutrition composition, specifically carbohydrate intake, on the incidence of RH in patients with AN [1]. To our knowledge, there are limited studies that have investigated the link between carbohydrate intake and RH in orally fed rather than enterally fed children and adolescents. As such, there are a range of different practices, with some centres choosing not to restrict carbohydrate hence introducing a more varied intake earlier, however it is not known if this is a safe practice. If carbohydrate intake does not need to be restricted in order to minimise the risks of RFS, this may promote both an earlier and a more normalised approach to eating and weight restoration.

## Method

### Aim

The aim of this pilot study was to compare a standard carbohydrate aggressive oral feeding protocol with a low carbohydrate protocol on the risk of RH in paediatric patients with AN admitted for medical stabilisation.

### Design

This study was a single centre randomised controlled trial. The study was approved by the Austin Health Human Research Ethics Committee (HREC/16/Austin/533) and registered with the Clinical Trials Registry (ACTRN12621000300875). Consent was obtained from both the child or adolescent and their guardian prior to participation in the study. A convenience sample was planned based on the number of admissions across the study period between September 2017 and October 2019.

### Setting

The Paediatric and Adolescent Inpatient Unit at the Austin Hospital in Melbourne, Australia, provides tertiary level inpatient care for children and adolescents with eating disorders who require medical stabilisation. Whilst recovery in the community is supported where possible, patients who are medically unstable are admitted for inpatient care. Criteria for inpatient admission includes bradycardia, systolic hypotension, orthostatic systolic hypotension, recurrent syncope, cardiac arrhythmia, ECG (electrocardiogram) abnormality, hypothermia, dehydration, electrolyte derangement, sustained rapid weight loss and/or severe malnutrition [13].

### Participants

Patients with AN or atypical AN as per the diagnostic criteria in the Diagnostic and Statistical Manual of Mental Disorders, Fifth Edition (DSM-5) [14], were included in the study. The eating disorder diagnosis was made by the Paediatrician. Patients were required to be 18 years of age or younger, expected to be an inpatient for a minimum of 7 days and managed under the hospital paediatric eating disorder protocol. Patients who presented with hypophosphatemia on admission, or who had transferred from another hospital or inpatient unit where nutritional rehabilitation had already been commenced, were excluded from the study.

### Anthropometry

Height and weight were measured at baseline and then twice weekly thereafter. Participants were weighed in a gown post void and prior to consuming breakfast. Anthropometric data were plotted on the Centers for Disease Control and Prevention growth charts [15]. Percent median BMI (%mBMI) was calculated This was calculated by current BMI/50th percentile BMI for age and sex × 100. Malnutrition status was assessed utilising BMI z score, percentage weight loss and %mBMI on presentation as per the Position Paper of the Society for Adolescent Health and Medicine: Medical Management of Restrictive Eating Disorders in Adolescence and Young Adults [13].

### Feeding protocol

The starting caloric prescription of the meal plan was assessed by the dietitian based on intake prior to admission following a comprehensive nutrition assessment, including anthropometric measures and malnutrition diagnosis. Participants deemed at high risk of RFS were those who who had minimal carbohydrate intake for 7–10 days. These participants were commenced on a meal plan of oral food and fluid providing 1500 kcal/day (6300 kJ). All other participants were commenced on a meal plan providing 2000—2500 kcal/d (8400–10,500 kJ). Participants were randomly assigned via concealed allocation to either a low carbohydrate feeding plan which provided less than 40% of total energy from carbohydrate, as per current practice, or a standard carbohydrate feeding plan which provided 50–60% of total energy from carbohydrate as per the Acceptable Macronutrient Distribution Ranges [16]. Caloric prescription for the two feeding plans was matched. Meal plans were increased incrementally by approximately 400 kcal twice weekly, until the participant reached a meal plan of 3000 kcal (12600 kJ). This was achieved by day 4–7 of admission (and by day 10 for those starting on 1500 kcal/day). Following this, increases to meal plans were dependent on adequacy of weight gain with the expectation of 1–1.5 kg weight gain per week as per local hospital guidelines. Once the participants reached the 3000 kcal meal plan there was no further difference in the carbohydrate content of the meal plans, with carbohydrates providing 50–60% total energy.

All meals and snacks were supervised and supported by nursing staff. If participants were unable to consume the entirety of their prescribed meal or snack they were required to have a nutritionally equivalent supplement drink or “bolus”. The supplement drink varied in carbohydrate composition in line with the treatment arm. The bolus was initially offered orally, however if the participant was unable to consume it orally it was administered via a nasogastric tube. Nutritional intake was documented on the fluid balance chart and reviewed by the dietitian twice weekly. Participants were on supervised bed rest following meal and snack times and bathroom visits were supervised.

### Medical monitoring

Participants were medically reviewed daily to monitor for clinical features of RFS including signs of congestive cardiac failure, confusion, and seizures.

Participants were monitored closely for biochemical markers of RFS, with analysis of electrolytes, calcium, magnesium, phosphate, glucose daily for 7 days and then twice weekly thereafter. In those patients deemed at high risk for RFS, biochemical markers were evaluated daily for 10 days and twice weekly thereafter during the admission.

Prophylactic phosphate was not routinely administered. Hypophosphatemia was diagnosed when serum phosphate levels were < 0.95 mmol/L in patients under 16 years and < 0.87 mmol/L in patients > 16 years as per local hospital guideline. Phosphate was prescribed in the form of Sandoz Phosphate 500 mg twice per day with titration as indicated if RH occurred. A general multivitamin was not provided to participants.

The medical parameters monitored during the study are outlined in Table 1.

**Table 1:** Medical monitoring of study participants

Day	Bloods	Clinical
1 (Baseline)	FBE UEC CMP Glucose LFT’s TFT’s Vitamin D Active vitamin B12 and folate Iron studies Others as indicated (i.e. FSH, LH, estradiol/ testosterone)	Regular postural observations Temperature QID BGL ECG Overnight cardiac monitoring Medical assessment for signs of RFS
2–7	Daily: UEC CMP Glucose	Postural observations QID BGL ECG (Day 2 & 3) Overnight cardiac monitoring (Day 2) Medical assessment for signs of RFS
8 onwards (as per usual protocol)	Twice Weekly: UEC CMP Glucose	Postural observations Medical assessment for signs of RFS

FBE = full blood examination; UEC = urea electrolytes and creatinine; CMP = calcium, magnesium and phosphate ; LFT = liver function test; TFT = thyroid function test; FSH = follicle-stimulating hormone; LH = luteinizing hormone; BGL = blood glucose level; ECG = electrocardiogram; QID = four times a day; RFS= refeeding syndrome

### Data analysis

Data were collected throughout the participant’s admission including anthropometric, biochemical, clinical and dietary data. The primary outcome was the incidence of RH, as defined previously, within the first 7 days of nutritional rehabilitation. Additional analysis compared the changes in body weight across admission.

### Statistical analysis

Data are reported as the mean and standard deviation for quantitative factors. The incidence of RH over time and the relationship between malnutrition status and RH was assessed using two-way repeated measures, ANOVA. Differences in body weight over the time of admission between groups was assessed by unpaired t test. Study findings were assessed in terms of statistical significance via p values. Statistical analysis was completed using SPSS version 26.0 [17]. The feeding plans were analysed using Foodworks Professional v8.0 [18].

## Results

**Fig. 1:**
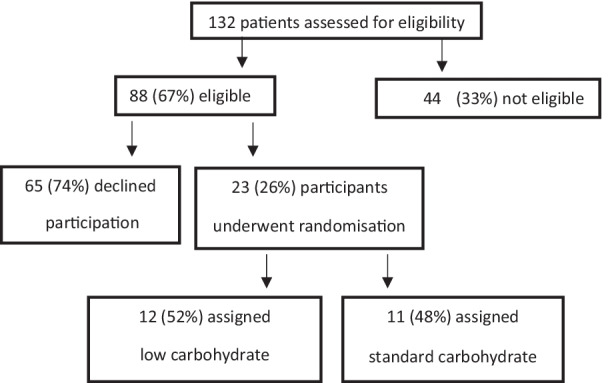
Flow diagram of eligible patients and random assignment to either low or standard carbohydrate group

Of the 132 eating disorder admissions during the 2-year study period, 67% (n = 88) were eligible as per the inclusion criteria. Of those eligible, 26% (n = 23) of patients and their parents/guardians provided consent to participate in the study (Fig. 1). Reasons for ineligibility included having a diagnosis other than AN or atypical AN (n = 9), commencing on a higher calorie intake for clinical reasons (n = 10), having already commenced nutritional rehabilitation in another setting prior to admission to the Austin paediatric ward (n = 10), being managed off the standard protocol for clinical reasons (n = 9), length of stay < 48 h (n = 5) and low phosphate on admission (n = 1).

Characteristics of the participants are outlined in Table 2.

**Table 2:** Baseline patient characteristics

	Low carbohydrate diet	Standard carbohydrate diet
Gender (M/F)	0/12	0/11
Age (years)	16.0 ± 1.3	14.4 ± 1.9
Days of admission	16.2 ± 6.1	19.4 ± 7.6
**Diagnosis**		
AN-Restricting, (n)	7	9
AN – Binge/purge, (n)	0	0
AN-Atypical, (n)	5	2
**Malnutrition diagnosis**		
Mild-Mod, n (%)	7 (58%)	9 (82%)
Severe n, (%)	5 (42%)	2 (20%)
BMI on admission (kg/m^2^)	19.2 ± 4.1	17.1 ± 2.5
BMI Z score on admission	−1.1 ± 2.1	−1.3 ± 1.1
%mBMI	92.4% ± 19	86.5% ± 10.5
**Prescribed energy intake on admission**		
1500 kcal (6300 kJ), n (%)	4 (33%)	5 (45%)
2000 kcal (8400 kJ), n (%)	7 (58%)	6 (55%)
2500 kcal (10500 kJ), n (%)	1 (8%)	0
Phosphate levels at baseline (mmol/L)	1.24 ± 0.12	1.27 ± 0.19

%mBMI = percent median BMI; Data presented as mean ± SD where indicated

The study protocol was adjusted to remove blood glucose level (BGL) monitoring as a mandatory requirement of participation due to the participants often declining testing due to fear of needles. As a result, there was insufficient information for meaningful data analysis and interpretation of the changes to blood glucose levels with refeeding in this study. Usual protocols within our unit do not include regular BGL monitoring unless clinically indicated.

The number of dairy serves as per the Australian Guide to Healthy Eating (AGHE) [19] provided in both the low and standard carbohydrate groups are outlined in Table 3.

**Table 3:** Comparison of dairy serves between the two diet groups as per AGHE

Prescribed energy intake on admission	Dairy Serves	
	Low carbohydrate diet	Standard carbohydrate diet
1500 kcal (6300 kJ)	3 serves	1 ½ serves
2000 kcal (8400 kJ)	4 ½–5 serves	3 ½–4 serves
2500 kcal (10500 kJ)	4 ½–6 serves	4 ½–6 serves

Only 8% (n = 2) of participants required a nasogastric tube to enterally administer nutrition throughout their admission due to food refusal. For both participants, enteral nutrition made up only 2% of their total nutritional intake across their admission. 33% (n = 4) of participants from the low carbohydrate group and 45% (n = 5) of participants from the high carbohydrate group had a portion of their intake in the form of a bolus due to being unable to eat the meal or snack. For both groups, this attributed to less than 10% of the total number of meals and snacks that came from nutrition supplements (bolus) rather than food during their admission.

**Fig. 2:**
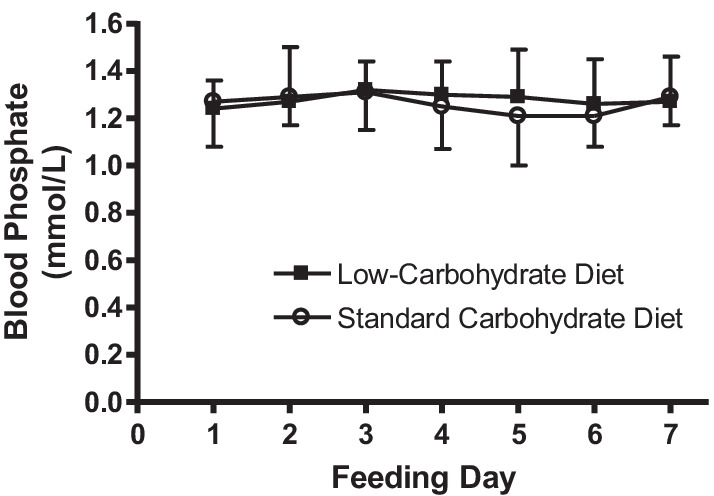
Change in blood phosphate levels over the initial week of admission. *Data presented as mean ± SD

Changes in phosphate levels over the time course of the feeding regimen are shown in Fig. 2. No participants developed RH throughout the first 7 days of admission. There was no significant effect of time on phosphate levels, or an interaction effect between either diet type or malnutrition status on phosphate levels over time. One participant in the standard carbohydrate group was given a single dose of phosphate supplementation on day 6 despite the participant’s phosphate levels being 0.96 mmol/L which was still in the normal range for patients under 16 years of age. This dose was prescribed by the medical team given a persistent downward trend.

Table 4 presents data on weight change over the first week of admission. Weight gain during the first week of admission was significantly higher in the standard carbohydrate group as was percentage point change in % median BMI from admission to discharge. However. total amount of weight gained over the entire admission was not significantly different between the two diet groups.

**Table 4:** Change in body weight over admission

	Low carbohydrate diet	Standard carbohydrate diet	*P* value
Weight gain first week (kg)	0.6 ± 0.9	1.4 ± 0.5	0.03
Total weight gain over admission (kg)	2.3 ± 2.0	3.5 ± 1.3	0.12
Average weight gain/week (kg)	0.8 ± 0.8	1.2 ± 0.3	0.15
% median BMI on discharge	96.3 ± 16.9	93.5 ± 11.0	0.65
% point change in % median BMI from admission to discharge	3.8 ± 3.3	7.0 ± 3.9	0.04

Data presented as mean ± SD

There was no clinically significant oedema reported in either of the diet groups. Aside from sinus bradycardia which would be expected due to the malnutrition, cardiac monitoring and ECG’s did not reveal significant electrophysiological abnormality in either group.

## Discussion

The findings of this pilot study support that a caloric equivalent low carbohydrate oral diet (< 40% total energy) commencing at a minimum of 1500 kcal/day has no advantage, compared to a standard carbohydrate oral diet (50–60% total energy) in reducing the incidence of RH and RFS in hospitalised patients with AN. Our study indicates that it is safe to provide higher calorie feeding without needing to initially restrict energy from carbohydrates, or needing to routinely supplement with prophylactic phosphate, which contrasts with previous studies [1, 11, 12, 20]. The refeeding risks in our study were able to be safely managed with medical monitoring and phosphate supplementation as clinically indicated.

Rapid introduction of carbohydrates following a period of inadequate food intake is thought to precipitate the electrolyte and fluid shifts that occur during RFS [2, 5, 20]. As the body shifts from a catabolic to an anabolic state, insulin levels increase which in turn increases glucose metabolism. The demand for phosphate during glucose carbohydrate metabolism is high and serum phosphate levels may fall, thereby increasing the risk of RH and RFS [20, 21]. Hence, in aggressive feeding protocols, restricting the amount of carbohydrates during the early stages of nutritional rehabilitation to reduce the demand for phosphate, and/or administering prophylactic phosphate supplementation to attenuate the risk of RH has been trialled, despite limited evidence supporting either practice [6, 7].

The serum phosphate levels did not decrease below range for any of the participants in either the low or standard carbohydrate groups in our study. One participant was cautiously given a dose of oral phosphate in the setting of a downward trend in serum phosphate without clinical markers of evolving RFS. Without this supplementation, it is unknown if the participant’s serum phosphate level may have stabilised on its own. The results of our study suggest that with regular clinical and biochemical monitoring, the risks of RFS can be safely mitigated without prophylactic phosphate replacement.

The MARSIPAN guidelines identify increasing dietary phosphorus (e.g. milk) as a strategy for avoiding RFS, although the recommended amount of dietary phosphorus to be included is not outlined [7]. Dietary intake of the micronutrient phosphorus directly impacts serum phosphate levels [22]. Milk and dairy products are the food group which contain the greatest amount of dietary phosphorus [23]. To ensure caloric equivalence between the two diet groups whilst manipulating the percentage carbohydrate, the number of dairy serves, as per the AGHE [19], in the standard carbohydrate was less than in the low carbohydrate group until 2500 kcal/day. In our study, despite the potential for the standard carbohydrate group to be at an increased risk of RH due to the higher carbohydrate load and the lower content of dietary phosphorus, there was no increased risk of RH.

A major difference between oral and enteral feeding is the consistency of macronutrient distribution that is provided by the formulas used for enteral feeding. The majority of standard nutrition supplements provide ≥ 50% energy from carbohydrate [24]. It is thought that the risk of RFS may be greater with enteral or parenteral feeding compared to oral feeding [25]. In the instance of enteral feeding, the potential benefits of continuous feeding to reduce the risk of RFS are outlined by Kohn and Madden [21]. It is likely that this is because continuous feeding provides a consistent and regulated carbohydrate supply. However, despite this there is little evidence to support these recommendations. In Hale and Logomarsino’s systematic review of 1665 patients treated with enteral nutrition, RH and mild oedema which are precursors to RFS, were reported in 41% of the studies [26]. They conclude that despite this there was no significant difference between RFS, or electrolyte abnormalities in enterally fed cohorts compared to orally fed cohorts [25]. In many studies looking at the safety of aggressive feeding protocols, the percentage of energy from carbohydrate is not described. Whilst inpatient eating disorder treatment may take a range of different approaches, our unit advocates for a food-based rather than supplement-based approach. A key feature of our study was the use of an oral nutritional rehabilitation protocol and our low use of nutritional supplements (< 10% total meals/snacks). To our knowledge, there have been no studies that investigate the difference between the risk of RH in AN between purely food based oral diet compared to those that rely more heavily on nutrition supplements administered either orally or enterally. Therefore, our results could not necessarily be extrapolated to patients receiving a majority of their nutrition from nutrition supplements.

Our study focused specifically on the incidence of RH in children and adolescents with AN. Many of the studies that support aggressive feeding approaches do so specifically in a child and adolescent population [11, 12]. Children and adolescents with AN tend to present for treatment earlier in the course of the illness than adults, as their guardians often notice the early signs and symptoms of the disorder and seek help [27]. Therefore, it is common that adults with AN present at later stages in their illness, and in a more severely and chronically malnourished state. There are no evidence-based guidelines for the re-introduction of nutrition in children with an eating disorder [28]. The Junior MARSIPAN guidelines do not outline clear recommendations regarding the starting caloric intake for children, however acknowledge the emerging evidence to support aggressive re-feeding [11, 28]. The recommendations outlined in the RANZCP guidelines [5] recommend starting adults at ~ 1400 kcal/day (6000 kJ), and those in the MARSIPAN guidelines [6] recommend commencing adults at 15–20 kcal/kg/day. However, for patients at high risk of RFS, it is advised to start caloric intake at 5–15 kcal/kg/day with progressive increases to 20 kcal/kg/day within 2 days provided there are no contraindications to increasing. Given the seemingly higher risk of RFS in adult patients it cannot be assumed that a standard carbohydrate intake of (50–60% total energy) is safe to provide adult patients hospitalised with AN. This area requires further study.

Patients with atypical AN are often not included in refeeding studies. It has been identified that the degree of malnutrition, including % weight loss, and the avoidance of dietary carbohydrates rather than BMI alone increases the risk of RFS [4]. For this reason, patients with a diagnosis of atypical AN were included in our study and contributed to 30% of our participants. The medical and psychiatric complications of patients with atypical AN are like those seen in patients with AN, with patients frequently losing a large amount of weight very rapidly through engaging in eating disorder behaviours including restriction and over-exercising [29]. Therefore, it is important to consider patients with atypical AN as being at risk of RFS based on degree of malnutrition and clinical assessment, rather than the eating disorder diagnosis alone.

Our results indicate weight gain in the low carbohydrate group was significantly lower across the first week of admission. Weight restoration of ≥ 0.8 kg/week during nutritional rehabilitation is associated with improved outcomes post discharge [10]. In our study, weight restoration of o.6 kg/week in the first 7 days in the low carbohydrate group, compared to 1.4 kg/week in the standard carbohydrate group, indicates that a standard carbohydrate diet supports maximal weight gain in the shortest length of stay. During starvation, glycogen stores from the liver and muscle are mobilised, and gluconeogenesis acts to maintain neurological function [30]. When nutrition is available in sufficient quantities, stores of both glycogen and water must replete [30] and weight subsequently increases [7]. The standard carbohydrate group achieved greater weight gain during the first week of admission, however average weight gain across the entire admission did not differ between the two groups. It is possible that the observed difference was due to a greater degree of early subclinical fluid retention in the standard carbohydrate group, and that this fluid had dissipated by the time of discharge [31]. Given the relationship between fluid shifts and development of RFS, this reinforces the need to closely monitor these patients for any biochemical and clinical signs of RFS to enable corrective action to be taken with supplementation as required.

It should also be noted that among the studies looking at RH, there are varying thresholds for diagnosis and treatment of hypophosphatemia, thereby limiting comparison between units.

In our study, we were unable to commence all participants on the same caloric intake, needing to consider their pre-admission intake, degree of malnutrition and risk of RFS. Despite the different caloric prescription of the meal plans, the percentage of energy coming from carbohydrates was consistent across all meal plans (< 40% of total energy from carbohydrate in the control arm and 50–60% of total energy from carbohydrate in the treatment arm). Although we qualitatively analysed the participant’s pre-admission intake, a limitation of this study was that we did not complete a quantitative dietary assessment. This would have provided us with more detailed information on the participant’s macro and micro nutrient distribution between home and hospital intake.

We acknowledge the small sample size of this pilot study. Recruitment posed several challenges. Many of our young patients with eating disorders feared that a higher carbohydrate intake would result in greater long-term weight gain. Consequently, many children and adolescents declined participation despite their parents and guardians providing consent. We accept considerable numbers are required to determine a correlation between carbohydrate intake, RH and RFS, given the low incidence of clinically significant RH in malnourished populations with AN [4, 32]. Nevertheless, the results from this pilot study provide our unit with the confidence to implement a standard carbohydrate intake (50–60% total energy) as usual practice. Ongoing data from this practice will continue to be monitored and evaluated for safety and efficacy. Once implemented, this will enable retrospective data to be collected and a larger population to recruit from to affirm the results of our pilot study.

Once allocated to either the low or standard carbohydrate diet groups, participants were no longer blinded from the researchers and although participants were not informed of which diet group they were allocated to, it was evident from the types of food items provided which diet group the participants were allocated to. We do not believe that this would have had an impact on our results given participants did not have access to any food other than that provided by their meal plans.

## Conclusion

The results of this pilot study suggest that a standard carbohydrate diet providing 50–60% of total energy from carbohydrate, does not increase the risk of hypophosphatemia despite an aggressive oral feeding protocol in child and adolescent inpatients with AN. This study suggests that low carbohydrate refeeding protocols result in slower weight restoration across the first 7 days of admission. We are aware that with the low incidence of RFS, there needs to be studies in larger populations to obtain conclusive evidence. The results of this pilot study support the need to conduct a larger, multicentre randomised control trial to provide definitive evidence of the safety and benefits of standard carbohydrate intake in nutritional rehabilitation of children and adolescents with AN.

## Data Availability

The datasets used and/or analysed during the current study are available from the corresponding author on reasonable request.

## Abbreviations

AN: Anorexia nervosa
RFS: Refeeding syndrome
RH: Refeeding hypophosphatemia
kcal: Kilocalories
%mBMI: Percent median BMI
BGL: Blood glucose level
ECG: Electrocardiogram
AGHE: Australian Guide to Healthy Eating

## References

[1] Garber A, Sawyer S, Golden N, Guarda A, Katzman D, Kohn M (2016). A systematic review of approaches to refeeding in patients with anorexia nervosa. Int J Eat Disord.

[2] Friedli N, Stanga Z, Culkin A, Crook M, Laviano A, Sobotka L (2018). Management and prevention of refeeding syndrome in medical inpatients: an evidenced-based and consensus-supported alogorithim. Nutrition.

[3] Boatend A, Sriram K, Meguid M, Crook M (2010). Refeeding syndrome: treatment considerations based on collective analysis of literature case reports. Nutrition.

[4] O’Connor G, Nicholls D (2013). Refeeding hypophosphatemia in adolescents with anorexia nervosa: a systematic review. Nutr Clin Pract.

[5] Friedeli N, Odermatt J, Reber E, Schuetz P, Stanga Z (2020). Refeeding syndrome: update and clinical advice for prevention, diagnosis and treatment. Curr Opin Gastroenterol.

[6] Hay P, Chinn D, Forbes D, Madden S, Newton R, Sugenor L et al. The Royal Australian and New Zealand College of Psychiatrists Clinical practice guidelines for the treatment of eating disorders. Australian and New Zealand. Aust NZ J Psychiatry. 2014;48(11):977–1008. 10.1177/0004867414555814 25351912

[7] Robinson P, Dahabra S, Morgan J, Nicholls D, Sharma S, Winston A. MARSIPAN: Management of Really Sick Patients with Anorexia Nervosa. 2nd edition. Royal College Psychiatrists and Royal College Physicians London. College Report CR162. 2010.

[8] Sylvester C, Forman S (2008). Clinical practice guidelines for treating restrictive eating disorder patients during medical hospitalization. Curr Opin Pediatr.

[9] Katzman DK (2005). Medical complications in adolescents with anorexia nervosa: a review of the literature. Int J Eat Disord.

[10] Lund B, Hernandez E, Yates W, Mitchell J, McKee P, Johnson C (2009). Rate of inpatient weight restoration predicts outcome in anorexia nervosa. Int J Eat Disord.

[11] Whitelaw M, Gilverston H, Lam P, Sawyer S (2010). Does aggressive refeeding in hospitalized adolescents with anorexia nervosa result in increased hypophosphatemia?. J Adolesc Health.

[12] Madden S, Miskovic-Wheatley, Clarker S, Touyz S, Hay P, Kohn MR. Outcomes of a rapid refeeding protocol in adolescent anorexia nervosa. J Eat Disord. 2015;3:8. 10.1186/s40337-015-0047-1 PMC4379764 25830024

[13] The Society for Adolescent Health and Medicine (2015). Position paper of the society for adolescent health and medicine: medical management of restrictive eating disorders in adolescents and young adults. J Adolesc Health.

[14] American Psychiatric Association. Diagnostic and Statistical Manual of Mental Disorders (DSM-5). 5th edition. VA: Arlington; 2013.

[15] CDC growth charts: United States. [Internet]. Available from: http://www.cdc.gv/growthcharts/. Accessed Dec 2020.

[16] Dietary energy. In: Nutrient Reference Values for Australia and New Zealand [Internet]. Canberra ACT: Department of Health; 2006. http://www.nrv.gov.au. Accessed Dec 2020.

[17] SPSS Inc. Released 2019. SPSS Statistics for Windows, Version 26.0. Armonk, NY:IBM Corp.

[18] Foodworks 8 Professional, v8.0. Brisbane: Xyris Pty Ltd, 2015.

[19] Australian Guide to Healthy Eating. https://www.eatforhealth.gov.au/food-essentials/five-food-groups/milk-yoghurt-cheese-andor-their-alternatives-mostly-reduced-fat. Accessed December 2020.

[20] De Silva A, Nightingale JMD (2020). Refeeding syndrome: physiological background and practical management. Frontline Gastroenterol.

[21] Kohn M, Madden S, Clarke S (2011). Refeeding in anorexia nervosa: increased safety and efficiency through understanding the pathophysiology of protein calorie malnutrition. Curr Opin Pediatr.

[22] Phosphorus. In: Nutrient Reference Values for Australia and New Zealand [Internet]. Canberra ACT: Department of Health; 2006. http://www.nrv.gov.au. Accessed Dec 2020.

[23] Gutierrez O, Kalantar-Zadeh K, Mehrotra R (2017). Clinical Aspects of Natural and Added Phosphorus in foods.

[24] Nutricia Advanced Medical Nutrition. [Internet]. http://www.nutriciamedical.com.au. Accessed Dec 2020.

[25] Reber E, Friedli N, Vasiloglou MF, Schuetz P, Stanga Z. Management of refeeding syndrome in medical inpatients. J Clin Med. 2019;8(12)2202. 10.3390/jcm8122202 PMC6947262 31847205

[26] Hale M, Logomarsino J (2019). The use of enteral nutrition in the treatment of eating disorders: a systematic review. Eat Weight Disord.

[27] Johnston A, Morris J, McKinlay A (2018). Eating and feeding disorders in children and adolescents. Multidisciplinary management of eating disorders.

[28] Royal College of Psychiatrists. Junior MARSIPAN: Management of Really Sick Patients under 18 with Anorexia Nervosa. Royal College of Psychiatrists London. College Report CR168. 2012.

[29] Moskowitz L, Weiselbery E (2017). Anorexia nervosa/atypical anorexia nervosa. Curr Probl Pediatr Adolesc Health Care.

[30] O’Keefe S. Physiology of Human Nutrition: Starvation and Obesity. In: O’Keefe S. The principles and practice of nutrition support. New York:Springer Science; p 9–18.

[31] Marzola E, Nasser JA, Hashim SA, Shih PB, Kaye WH (2013). Nutritional rehabilitation in anorexia nervosa: review of the literature and implications for treatment. BMC Psychiatry.

[32] Ornstein R, Golden N, Jacobson M, Shenker I (2003). Hypophosphatemia during nutritional rehabilitation in anorexia nervosa: implications for refeeding and monitoring. J Adolesc Health.

